# Improving Mental Health Knowledge and Reducing Mental Health Stigma Among Public Safety Personnel: Comparison of Live vs. Online Psychoeducation Training Programs

**DOI:** 10.3390/ijerph21101358

**Published:** 2024-10-15

**Authors:** Madeline R. Marks, Clint Bowers, Deborah C. Beidel, Jordan Ortman, Amie R. Newins

**Affiliations:** 1 Department of Psychiatry, School of Medicine, University of Maryland-Baltimore, Balitmore, MD 21201, USA; 2 Department of Psychology, College of Sciences, University of Central Florid, Orlando, FL 32816, USA; clint.bowers@ucf.edu (C.B.); deborah.beidel@ucf.edu (D.C.B.); jordan.ortman@ucf.edu (J.O.); amie.newins@ucf.edu (A.R.N.)

**Keywords:** public safety personnel, first responders, psychoeducation, stigma, stress, brief interventions

## Abstract

This study evaluates the effectiveness of a brief psychoeducation training program in reducing mental health stigma, both toward others and oneself, among public safety personnel, while also comparing the relative effectiveness of in-person and online training modalities. In total, 1686 public safety personnel in Florida received psychoeducation on the mental health impacts of public safety work. Participants completed pre- and post-training questionnaires assessing demographics, mental health knowledge, and mental health stigma toward others and themselves. Among the participants, 871 completed the training online, and 44 completed the training in-person. A paired samples *t*-test assessed changes in knowledge from pre- to post-test, and 2 × 2 repeated measures ANOVAs analyzed stigma-related data. Mental health knowledge increased and mental health stigma toward others decreased post-training, with no differences between training modalities. No changes in mental health self-stigma were found. Police officers reported significantly greater self-stigma than firefighters. Firefighters and dispatchers reported significantly less stigma toward others than police officers. This study found that both in-person and online psychoeducation can similarly improve mental health knowledge and reduce stigma toward others, which may help reduce barriers to seeking care.

## 1. Introduction

Public safety personnel, such as law enforcement officers, firefighters, and emergency dispatchers are frequently exposed to significant stressors including physical danger, environmental discomfort, time pressure, and traumatic events [1]. These stressors contribute to a high prevalence of negative mental health outcomes among this group such as posttraumatic stress disorder (PTSD), anxiety disorders, substance misuse, depression, and suicide [2,3,4]. Furthermore, these mental health challenges often extend beyond the workplace, negatively impacting family relationships and leading to additional stress due to a lack of critical support systems [5,6].

Despite the high levels of mental health risk, public safety personnel underutilize mental health services, a trend driven by both organizational barriers and mental health stigma. Organizational barriers include difficulties in accessing providers and taking time off for treatment [7]. Additionally, mental health stigma, defined as the negative beliefs and attitudes individuals hold toward mental health problems and treatment, is highly prevalent among public safety personnel [8,9]. This stigma may lead to avoidance of treatment and worsening of their symptoms [10,11]. The strong workplace culture among public safety personnel, which often emphasizes strength and self-reliance, reinforces these stigmatizing attitudes. Furthermore, traditional masculine sex roles associated with these professions [12] contribute to the perception that admitting to mental health problems is a sign of weakness or laziness [8], potentially leading to discrimination or punishment by co-workers or employers [13].

Recent research has differentiated between two distinct forms of stigma: stigma toward others (public stigma) and self-stigma. Public stigma includes negative attitudes toward co-workers with mental health problems, often reflecting perceived societal views of mental health [14]. High levels of public stigma may lead to distrust and reluctance to work with individuals known or suspected to have mental health problems. Conversely, self-stigma refers to how individuals perceive themselves in light of mental health problems or treatment, which can result in low self-esteem and hesitancy to seek treatment [15].

Given that mental health stigma impacts how individuals with mental health problems are treated by others and the decision to seek treatment [16,17,18], it is important to develop and implement effective stigma-reducing programs. Psychoeducation programs designed to disseminate accurate knowledge about common mental health conditions, their underpinnings, and available treatments might help challenge harmful stereotypes regarding individuals with mental health conditions, which may help reduce mental health stigma. Such programs have demonstrated efficacy in reducing stigma across various populations [17,18,19]. Public safety personnel, a large and widely dispersed group, can particularly benefit from psychoeducation programs delivered via online platforms, as this modality is cost-effective, accessible across agencies, and aligns with mandatory continuing education requirements. However, research indicates that psychoeducation programs yield significant, but relatively small, effects on stigma [10,17,20]. Many existing programs are lengthy and resource-intensive, such as Mental Health First Aid, which involves 12 h of content [21], and the Road to Mental Recovery program, which is a one- to three-day program [22]. These lengthy, in-person formats may not be feasible for smaller or volunteer-based departments with limited budgets and staffing. Moreover, the available data suggests that shorter interventions could achieve similar outcomes without imposing significant time demands [20].

There is a need for brief, scalable, and effective psychoeducation programs for public safety personnel. It is essential to assess whether brief psychoeducation programs, particularly those delivered via an online modality, can enhance overall knowledge of mental health problems and decrease public and self-stigma. Given the unique stressors and workplace culture within public safety professions, this research holds significant implications for improving access to mental health care and fosters supportive environments for public safety personnel facing mental health challenges. Therefore, the purpose of the present study was to evaluate the effectiveness of a brief psychoeducation training program in improving mental health knowledge and reducing both public stigma and self-stigma toward mental health among public safety personnel. Additionally, the study aimed to compare the relative effectiveness of in-person versus online training modalities. There were two specific hypotheses:

1. The brief psychoeducational training would improve knowledge of mental health problems among public safety personnel.
2. The brief psychoeducation training would reduce mental health stigma toward others and oneself among public safety personnel.

In addition, exploratory analyses examined whether the effectiveness of the training differed by modality (i.e., in-person vs. online).

## 2. Materials and Methods

### 2.1. Participants and Procedures

The participants were public safety personnel in Florida recruited as part of a training initiative in response to a state law requiring public safety personnel to complete training in mental health awareness. Individuals who attended the training program offered by the authors’ organization were invited to participate in the research study. The study used a two-group (face-to-face vs. virtual) pre-post quasi-experimental design. Participants were provided a link to a Qualtrics survey about knowledge and stigma both before the start of the training and immediately following the completion of the training. A consent form describing the research study was included on the first page of the pre-training survey. All data were collected in accordance with American Psychological Association Guidelines. The protocol was approved by the University of Central Florida Institutional Review Board.

### 2.2. Training Program

The training program was developed by a licensed psychologist who serves as the executive director of UCF RESTORES, a clinical research center specializing in the identification and treatment of trauma-related disorders at the University of Central Florida, and a doctoral student (first author). The one-hour training comprised four modules: Trauma; Responses to Critical Incidents; When Symptoms Become a Disorder; and Treatment and Resources. The training began with information about job-related stressors, how stress impacts physical, cognitive, and emotional functioning, and when too much stress can lead to mental health challenges. This information was followed by a description of common mental health problems among first responders (e.g., PTSD, depression, anxiety, anger, substance misuse, sleep problems) and coping strategies to help reduce mental health symptoms (e.g., sleep hygiene, relaxation strategies). Next, the training provided recommendations for choosing a mental health clinician. The in-person version of the training was delivered by doctoral students in clinical psychology using PowerPoint lectures. These students received training from the program developer on the content and delivery of the program material. The trainers facilitated discussion and points of inquiry during the lecture. The online version of the training was created as a video presentation. Each online video training began and concluded with either a fire chief or police chief, who acknowledged the stress of the job and emphasized the ideas that “it is OK not to be OK” and that “doing your job means taking care of yourself”.

### 2.3. Measures

#### 2.3.1. Mental Health Knowledge

The researchers created a 10-item knowledge test to assess participants’ learning of the presented material. Items were developed based on the content of the training program. The test items are included in the Supplementary Materials.

#### 2.3.2. Mental Health Stigma

Mental health stigma toward others and self-stigma were assessed using the Police Officer’s Stigma Scale (POSS) [23]. The POSS is an eleven-item scale designed to measure stigma among police officers. The items on the POSS demonstrate good internal consistency (α = 0.82), implying good reliability [23]. For the current study, the POSS was modified for firefighters and dispatchers (e.g., replacing “officer” with “firefighter”), resulting in three versions specific for each of the professions included in the sample. A recent study found that this change did not affect the scale’s psychometric properties [16]. Additionally, since the original POSS targets stigma toward others, items were modified by altering the stem to indicate feelings toward oneself to assess self-stigma. Higher scores on the POSS indicate lower levels of stigma.

### 2.4. Data Analysis

The Statistical Package for Social Sciences (SPSS, v.26) was used for subsequent data analysis. All tests were two-tailed unless otherwise stated, and results were considered statistically significant if the *p*-value was <0.05. Two-way repeated measures analyses of variance (ANOVAs) were used to test changes in knowledge and stigma from pre- to post-training and the effect of training modality (in-person vs. online). Two-way repeated measures ANOVAs were also used to test whether knowledge, stigma, or changes in these variables following training varied by type of public safety personnel. Preliminary analyses indicated that assumptions for the use of parametric tests were not violated.

## 3. Results

A total of 1570 public safety personnel attended the online training, and 871 completed the pre- and post-training questionnaires (104 police officers, 727 firefighters, and 40 dispatchers). A total of 116 public safety personnel attended the training in-person, and 44 completed the pre- and post-training questionnaires (21 police officers, 22 firefighters, and 3 dispatchers). Of the participants who completed the questionnaires, 87% were male, 12% were female, and 1% were nonbinary. The mean age was 38.13 years, and 8.4% of participants indicated a lifetime diagnosis of a mental health diagnosis.

### 3.1. Knowledge Test

A paired-samples *t*-test indicated a significant increase in knowledge from pre-test (*M* = 3.81, *SD* = 1.68) to post-test (*M* = 4.25, *SD* = 1.99), *t*(1088) = 6.88, *p* < 0.01. There were no significant differences by modality or among the different professions.

### 3.2. Effects of Training and Training Modality on Mental Health Stigma

**Figure 1:**
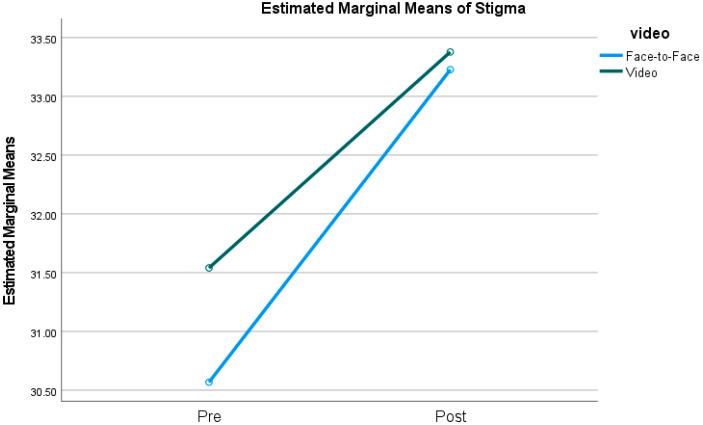
Pre- and post-training stigma toward others as a function of training modality.

When considering stigma toward others, the results of the ANOVA indicated a main effect of time (*F*(1,844) = 4.64, *p* < 0.05, eta^2^ = 0.005), such that mental health stigma improved from pre-training to post-training (*M_pre_* = 31.05, *SD_pre_* = 0.69, *M_post_* = 33.30, *SD_post_* = 0.79). There was no main effect for training modality (*F*(1,844) = 0.29, *p* = 0.69, eta^2^ = 0.00), and there was no interaction between time and training modality (*F*(1,844) = 0.15, *p* = 0.70, eta^2^ = 0.00), indicating that the change in stigma did not differ as a function of training modality. These results are illustrated in Figure 1.

### 3.3. Figure

For self-stigma, there were no significant main effects of time (*F*(1,844) = 3.64, *p* = 0.06, eta^2^ = 0.004) or training modality (*F*(1,844) = 1.82, *p* = 0.18, eta^2^ = 0.002). Additionally, there was no interaction between time and modality of delivery (*F*(1,844) = 1.27, *p* = 0.26, eta^2^ = 0.00).

### 3.4. Effects of Job Type on Mental Health Stigma

When considering stigma toward others, there was a main effect of job type (*F*(2,262) = 0.44, *p* ≤ 0.05, eta^2^ = 0.05). Least significant difference post hoc tests revealed that dispatchers (*M* = 36.03, *SD* = 1.11) and firefighters (*M* = 33.53, *SD* = 0.25) both reported significantly less stigma toward others compared to police officers (*M* = 29.30, *SD* = 0.69). There was no difference in the change from pre-training to post-training as a function of job type (*F*(2,626) = 0.43, *p* = 0.56, eta^2^ = 0.00.

For self-stigma, there was a main effect of job type such that police officers (*M* = 37.6, *SD* = 0.93 reported significantly greater levels of self-stigma than firefighters (*M* = 40.0, *SD* = 0.34, *F*(2,262) = 4.12, *p* < 0.05, eta^2^ = 0.008). No other group differences were statistically significant. There was no interaction between time and job type (*F*(2,613) = 0.65, *p* = 0.30, eta^2^ = 0.001).

## 4. Discussion

Mental health stigma is an important factor that may cause people to avoid seeking help for mental health problems and lead to worsening symptoms [11]. This concept might be particularly important for high-stress jobs such as those performed by public safety personnel as they are found to have a high prevalence of negative mental health outcomes [8,9]. Therefore, there is a need to explore trainings that facilitate the reduction of mental health stigma. Interestingly, because there seem to be differences between the professions, there may also be a need for future research to explore specific intervention strategies for different types of first responders.

The present study investigated the efficacy of a brief psychoeducation training program in improving mental health knowledge and reducing mental health stigma among public safety personnel, while also comparing the relative effectiveness of in-person and online training modalities. The results showed that the brief psychoeducation training program effectively increased knowledge about mental health among public safety personnel. The results also indicated that the training was associated with a small but statistically significant reduction in mental health stigma toward others, which did not differ by training modality (in-person vs. online). This finding is consistent with other studies that have shown that psychoeducation programs are effective in reducing stigma [19,20,21,22]. Although police officers reported higher levels of mental health stigma toward others, the training was equally effective for all groups.

The results indicated that the training did not influence mental health self-stigma. Although contrary to our expectations, this result is similar to those obtained by other psychoeducation programs [24,25,26,27]. However, recent research on self-stigma has demonstrated that it might be harder to reduce and may require more time-intensive training, such as actual exposure to individuals with mental health disorders [27]. However, since the scope of the problem is vast, it might be useful to use web-based programs to reduce stigma toward others while preserving resources for the more expensive training targeted at self-stigma.

Although the present study used a relatively large sample size, the study was still limited by a relatively small geographic dispersion, as all study participants were from the state of Florida, and results may differ by area. Future research using national samples would enhance the external validity of our findings. Additionally, exploring the long-term effectiveness and sustainability of psychoeducational training could provide further support for these programs as a valuable tool in reducing stigma. The persistence of self-stigma highlights the need for a deeper exploration of the unique factors contributing to this phenomenon within the public safety context. A better understanding of these factors would allow for the development of targeted self-stigma training. Furthermore, this study did not analyze the psychometric properties of the adapted POSS measure to compare to that found by those that adapted the measure for first responders [16]. Finally, the current study did not collect data on participants’ experiences of the training. Future research would benefit from examining acceptability data regarding psychoeducational training to address mental health awareness and stigma. Nonetheless, our results underscore the promise of online, video-based training as a scalable and cost-effective strategy for addressing certain dimensions of mental health stigma among public safety personnel.

## 5. Conclusions

In summary, the current study demonstrates that a brief video-based psychoeducation training program can effectively increase knowledge of mental health and reduce mental health stigma toward others. This format may be more viable and palatable for agencies with limited resources. Future research should investigate whether other trainings can be translated into a brief, technology-based format. Additionally, more research is required to understand and intervene with the more challenging issue of self-stigma.

## Data Availability

The data presented in this study are available upon request from the corresponding author due to privacy concerns.

## Supplementary Materials

The following supporting information can be downloaded at: https://www.mdpi.com/article/10.3390/ijerph21101358/s1, File S1: Pre-Training Knowledge Questionnaire.
