# Supplementary material for: Improving Mental Health Knowledge and Reducing Mental Health Stigma Among Public Safety Personnel: Comparison of Live vs. Online Psychoeducation Training Programs
Source: Int J Environ Res Public Health. 2024 Oct 15;21(10):1358. doi: 10.3390/ijerph21101358 (PMC11507392; doi:10.3390/ijerph21101358)
Supplement: Supplementary file 1 [file ijerph-21-01358-s001.zip › ijerph-3171007-supplementary.pdf]

Supplementary Materials: Pre-Training Knowledge Questionnaire

Q2 Under what circumstance is deep breathing a helpful mental health technique?

- Regulate anger
- Avoid thoughts and feeling
- Treatment for PTSD
- Improve memory and focus

Q3
A friend expresses feelings of hopelessness, isolation, deep sadness, and numbness. Based on this information, which of the following should you investigate further?

- PTSD
- Anger
- Depression
- Suicide

Q4 If I was interested in talking to someone about my mental health I should…

- Consult with a lawyer before further deciding what to do.
- Begin considering looking for a new job.
- Contact a confidential provider like UCF RESTORES or EAP.
- Wait and try to manage it on my own.

Q5 Most individuals will fall into the ____ zone(s) in response to stress.

- Orange/Red
- Yellow/Green
- Orange
- Red

Q6 Which of the following would you recommend to a friend that tells you they are having difficulty falling asleep?

- Drink alcohol
- Exercise before bed
- Watch T.V
- Set a sleep schedule

Q7 "I feel like a different person lately. I was always happy-go-lucky, make you laugh person, but now it seems like anything that anyone does is like nails on a chalkboard to me. I have not slept thru the night in over a month because of the nightmares. Sometimes I think I would feel so much better if I lived in the middle of the woods by myself or if I could punch anyone I want to in the face."

The above scenario best represents what stress zone?

- Red
- Yellow
- Orange
- Green

Q8 Three days after a bad call a friend is shouting, using a hateful tone, always has clenched fists, and low frustration tolerance. Together these are most likely signs that your friend is most likely experiencing the typical reaction of ______ following a trauma.

- Anger

- Depression
- PTSD
- Sleep problems

Q9 Which of the following would you recommend to a friend to do if they just experienced a traumatic event?

- Suggest they spend time alone.
- Tell them they have to talk about the event.
- Inform the person that exercise, especially outdoors, is often helpful.
- Recommend that they block the event out of their memory.

Q10 Your significant other is worried about a string of bad calls you have had. You let them know…

- Most people are diagnosed with PTSD immediately after a trauma
- Most people will not be diagnosed with PTSD after a trauma
- Most people will not be diagnosed if they have a good life
- Most people will be diagnosed with PTSD long after the trauma has occurred.

Q11 You should be concerned about someone's' drinking patterns if…

- They have 1 to 2 drinks per day with meals.
- They will only attend social events if there is alcohol.
- They choose not to drink.
- They collect and enjoy craft beer.
